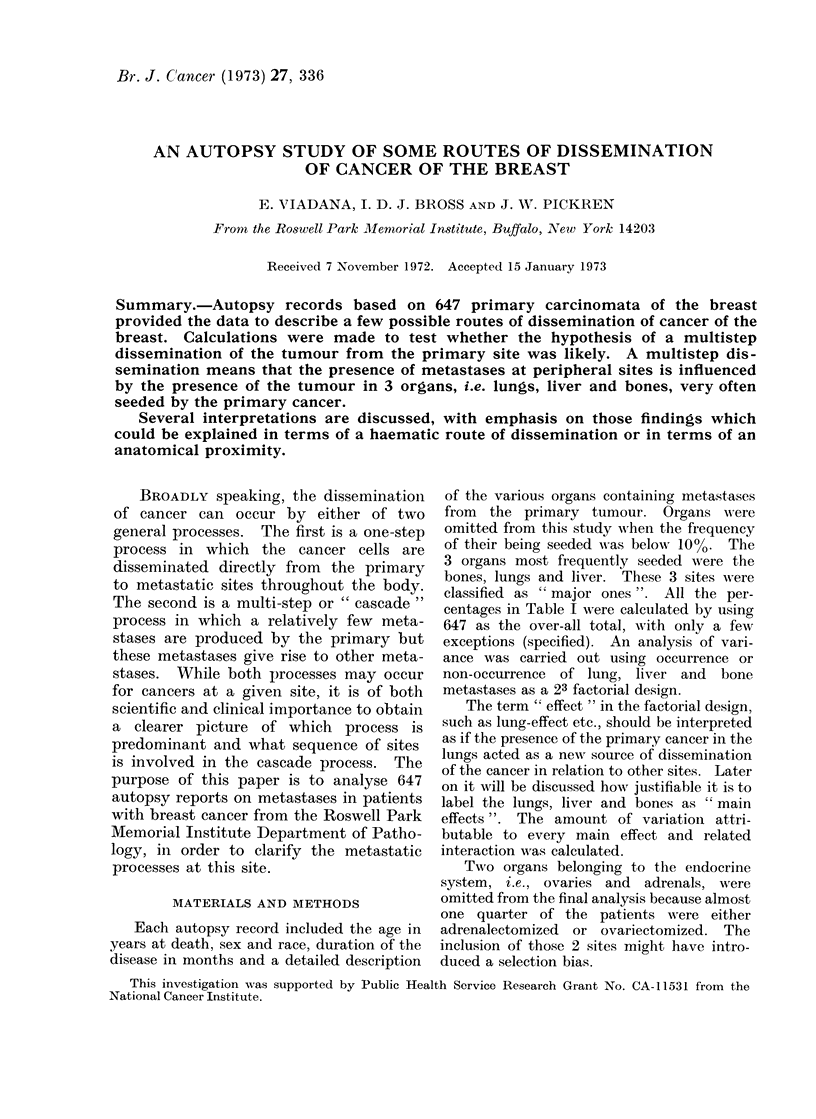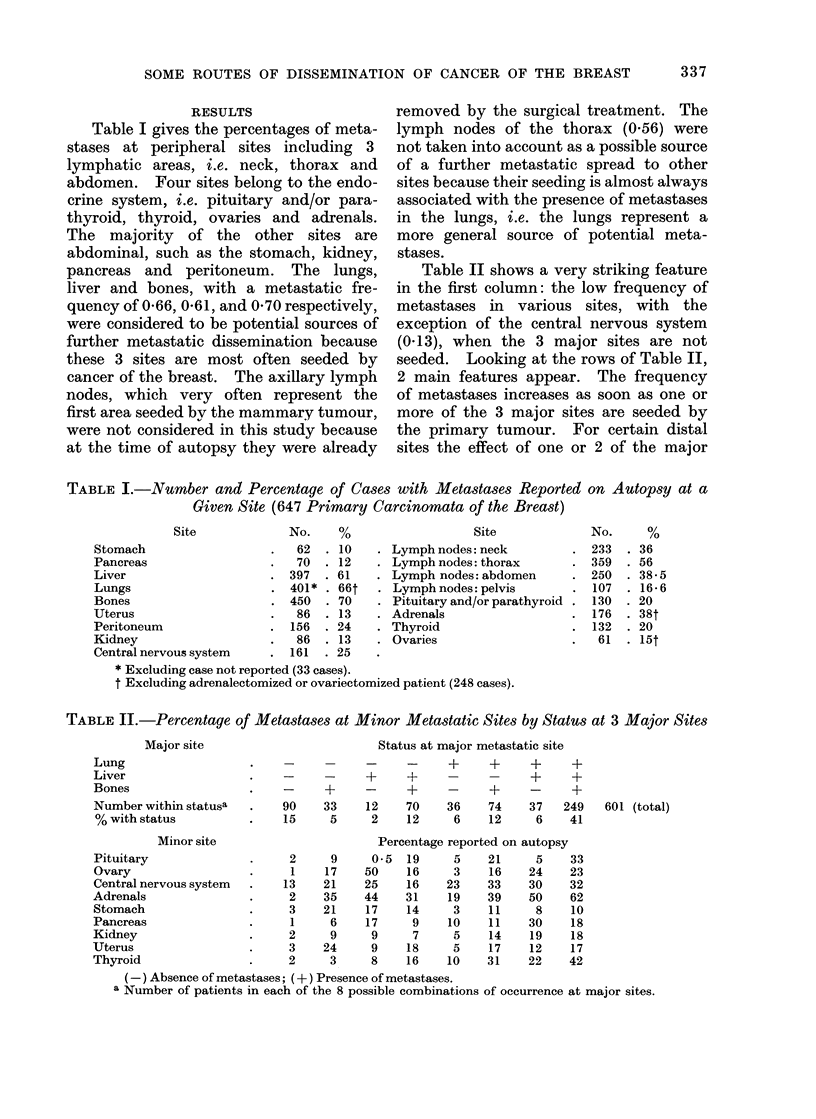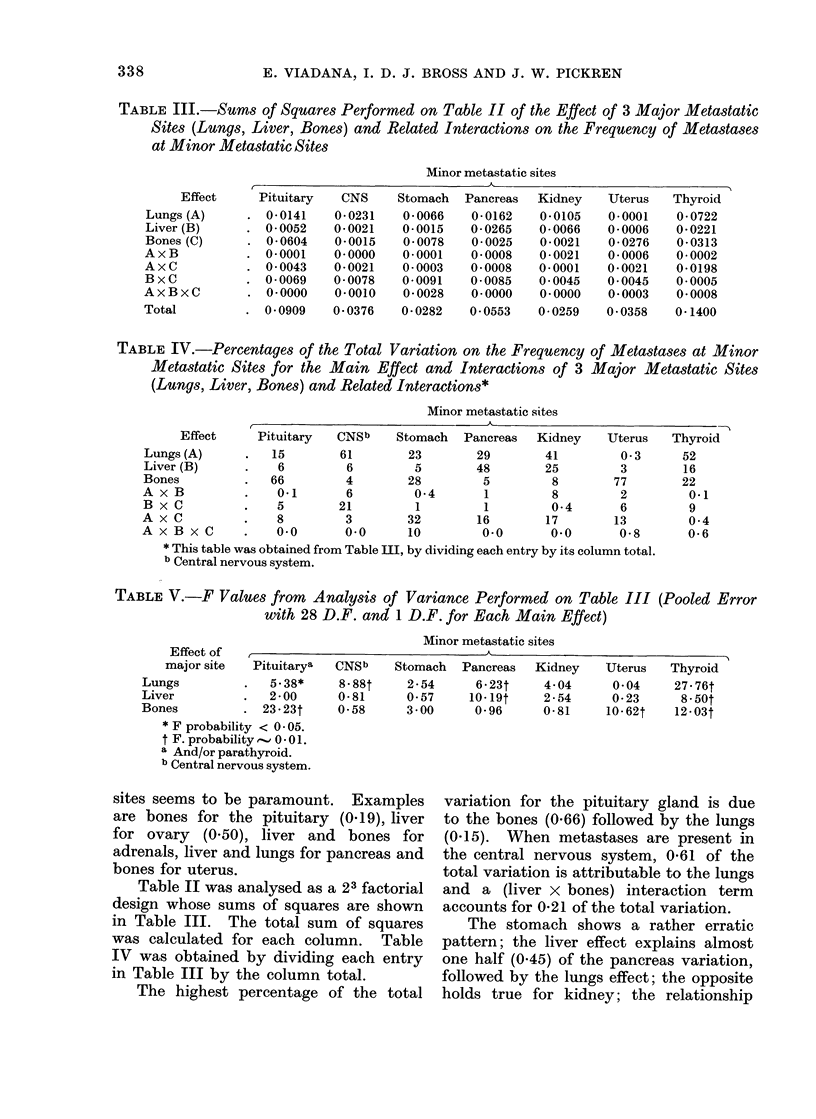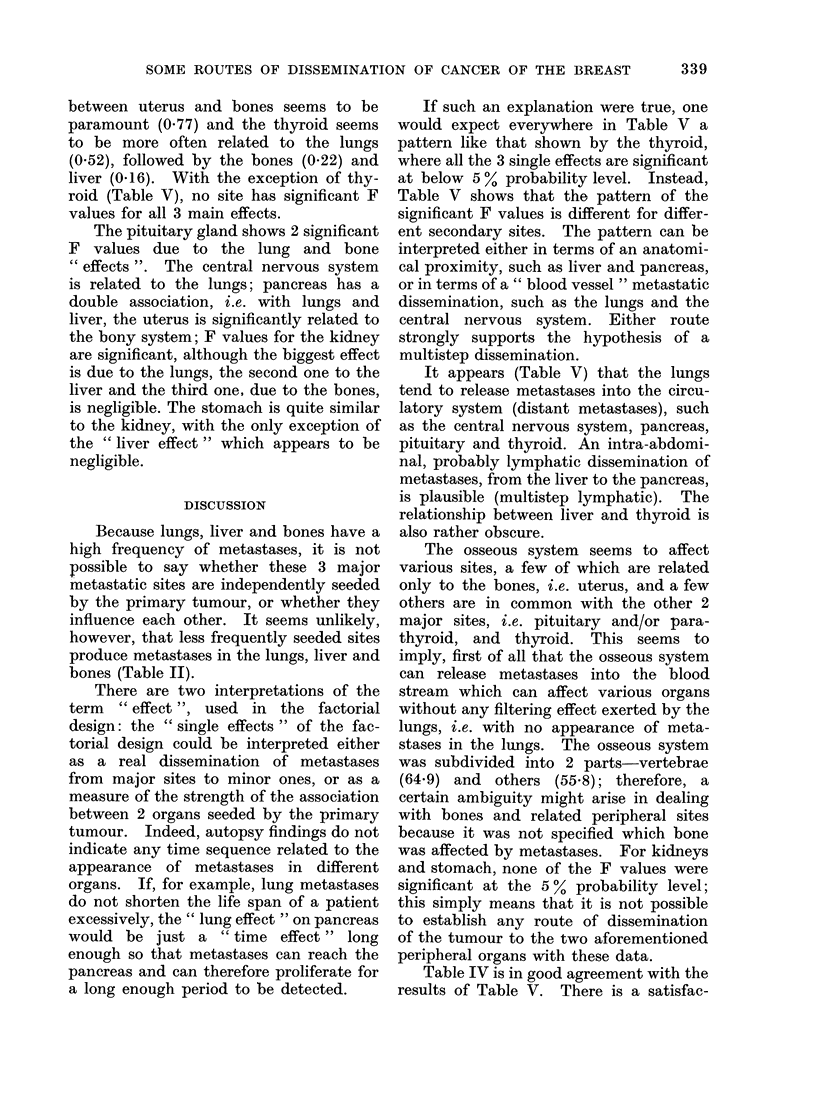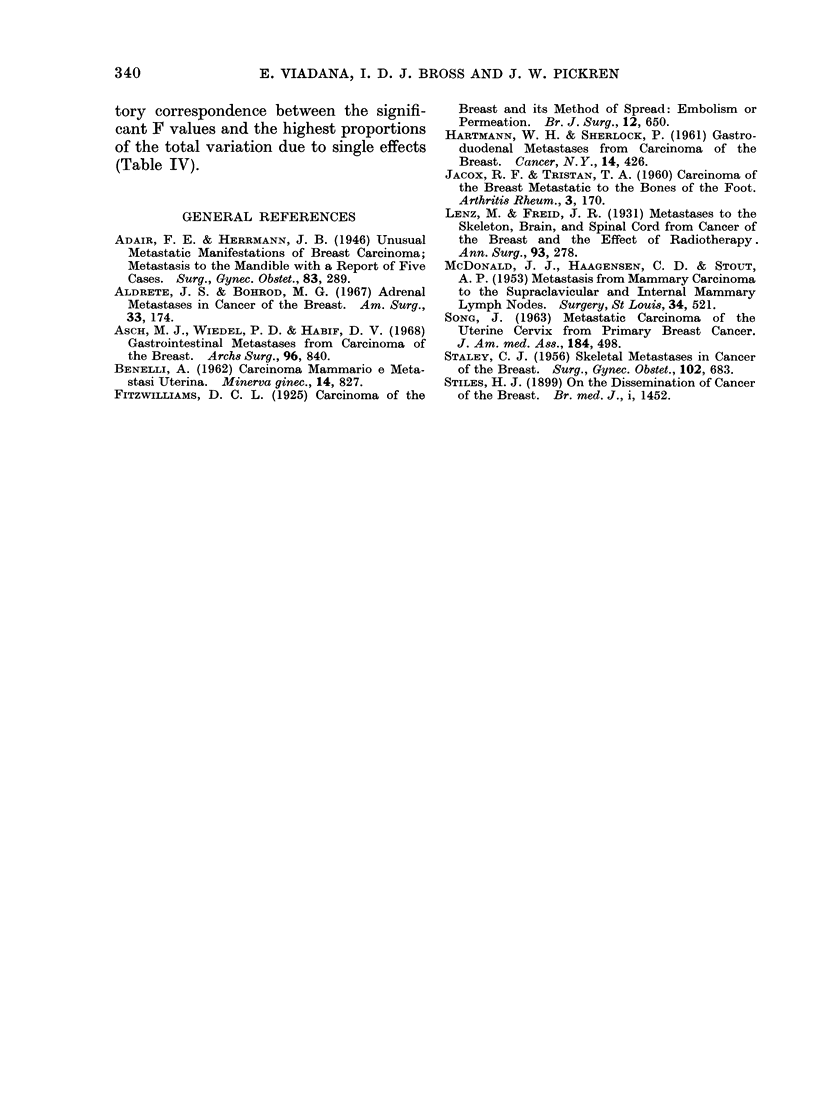# Am Autopsy Study of Some Routes of Dissemination of Cancer of the Breast

**DOI:** 10.1038/bjc.1973.40

**Published:** 1973-04

**Authors:** E. Viadana, I. D. J. Bross, J. W. Pickren

## Abstract

Autopsy records based on 647 primary carcinomata of the breast provided the data to describe a few possible routes of dissemination of cancer of the breast. Calculations were made to test whether the hypothesis of a multistep dissemination of the tumour from the primary site was likely. A multistep dissemination means that the presence of metastases at peripheral sites is influenced by the presence of the tumour in 3 organs, *i.e.* lungs, liver and bones, very often seeded by the primary cancer.

Several interpretations are discussed, with emphasis on those findings which could be explained in terms of a haematic route of dissemination or in terms of an anatomical proximity.


*Br. J. Cancer* (1973) **27**, 336

### AN AUTOPSY STUDY OF SOME ROUTES OF DISSEMINATION OF CANCER OF THE BREAST

E. VIADANA, I. D. J. BROSS AND J. W. PICKREN

*From the Roswell Park Memorial Institute, Buffalo, New York* 14203

Received 7 November 1972. Accepted 15 January 1973

**Summary.—Autopsy records based on 647 primary carcinomata of the breast provided the data to describe a few possible routes of dissemination of cancer of the breast. Calculations were made to test whether the hypothesis of a multistep dissemination of the tumour from the primary site was likely. A multistep dissemination means that the presence of metastases at peripheral sites is influenced by the presence of the tumour in 3 organs, *i.e.* lungs, liver and bones, very often seeded by the primary cancer.**

**Several interpretations are discussed, with emphasis on those findings which could be explained in terms of a haematic route of dissemination or in terms of an anatomical proximity.**

BROADLY speaking, the dissemination of cancer can occur by either of two general processes. The first is a one-step process in which the cancer cells are disseminated directly from the primary to metastatic sites throughout the body. The second is a multi-step or " cascade " process in which a relatively few metastases are produced by the primary but these metastases give rise to other metastases. While both processes may occur for cancers at a given site, it is of both scientific and clinical importance to obtain a clearer picture of which process is predominant and what sequence of sites is involved in the cascade process. The purpose of this paper is to analyse 647 autopsy reports on metastases in patients with breast cancer from the Roswell Park Memorial Institute Department of Pathology, in order to clarify the metastatic processes at this site.

### MATERIALS AND METHODS

Each autopsy record included the age in years at death, sex and race, duration of the disease in months and a detailed description of the various organs containing metastases from the primary tumour. Organs were omitted from this study when the frequency of their being seeded was below 10%. The 3 organs most frequently seeded were the bones, lungs and liver. These 3 sites were classified as " major ones ". All the percentages in Table I were calculated by using 647 as the over-all total, with only a few exceptions (specified). An analysis of variance was carried out using occurrence or non-occurrence of lung, liver and bone metastases as a $2^3$ factorial design.

The term " effect " in the factorial design, such as lung-effect etc., should be interpreted as if the presence of the primary cancer in the lungs acted as a new source of dissemination of the cancer in relation to other sites. Later on it will be discussed how justifiable it is to label the lungs, liver and bones as " main effects ". The amount of variation attributable to every main effect and related interaction was calculated.

Two organs belonging to the endocrine system, *i.e.*, ovaries and adrenals, were omitted from the final analysis because almost one quarter of the patients were either adrenalectomized or ovariectomized. The inclusion of those 2 sites might have introduced a selection bias.

This investigation was supported by Public Health Service Research Grant No. CA-11531 from the National Cancer Institute.

RESULTS

Table I gives the percentages of metastases at peripheral sites including 3 lymphatic areas, *i.e.* neck, thorax and abdomen. Four sites belong to the endocrine system, *i.e.* pituitary and/or parathyroid, thyroid, ovaries and adrenals. The majority of the other sites are abdominal, such as the stomach, kidney, pancreas and peritoneum. The lungs, liver and bones, with a metastatic frequency of 0·66, 0·61, and 0·70 respectively, were considered to be potential sources of further metastatic dissemination because these 3 sites are most often seeded by cancer of the breast. The axillary lymph nodes, which very often represent the first area seeded by the mammary tumour, were not considered in this study because at the time of autopsy they were already removed by the surgical treatment. The lymph nodes of the thorax (0·56) were not taken into account as a possible source of a further metastatic spread to other sites because their seeding is almost always associated with the presence of metastases in the lungs, *i.e.* the lungs represent a more general source of potential metastases.

Table II shows a very striking feature in the first column: the low frequency of metastases in various sites, with the exception of the central nervous system (0·13), when the 3 major sites are not seeded. Looking at the rows of Table II, 2 main features appear. The frequency of metastases increases as soon as one or more of the 3 major sites are seeded by the primary tumour. For certain distal sites the effect of one or 2 of the major

TABLE I.—*Number and Percentage of Cases with Metastases Reported on Autopsy at a Given Site* (647 *Primary Carcinomata of the Breast*)

| Site | No. | % | Site | No. | % |
|---|---|---|---|---|---|
| Stomach | 62 | 10 | Lymph nodes: neck | 233 | 36 |
| Pancreas | 70 | 12 | Lymph nodes: thorax | 359 | 56 |
| Liver | 397 | 61 | Lymph nodes: abdomen | 250 | 38·5 |
| Lungs | 401* | 66† | Lymph nodes: pelvis | 107 | 16·6 |
| Bones | 450 | 70 | Pituitary and/or parathyroid | 130 | 20 |
| Uterus | 86 | 13 | Adrenals | 176 | 38† |
| Peritoneum | 156 | 24 | Thyroid | 132 | 20 |
| Kidney | 86 | 13 | Ovaries | 61 | 15† |
| Central nervous system | 161 | 25 | | | |

* Excluding case not reported (33 cases).
† Excluding adrenalectomized or ovariectomized patient (248 cases).

TABLE II.—*Percentage of Metastases at Minor Metastatic Sites by Status at* 3 *Major Sites*

| Major site | Status at major metastatic site | | | | | | | | |
|---|---|---|---|---|---|---|---|---|---|
| Lung | – | – | – | – | + | + | + | + | |
| Liver | – | – | + | + | – | – | + | + | |
| Bones | – | + | – | + | – | + | – | + | |
| Number within status[a] | 90 | 33 | 12 | 70 | 36 | 74 | 37 | 249 | 601 (total) |
| % with status | 15 | 5 | 2 | 12 | 6 | 12 | 6 | 41 | |
| **Minor site** | **Percentage reported on autopsy** | | | | | | | | |
| Pituitary | 2 | 9 | 0·5 | 19 | 5 | 21 | 5 | 33 | |
| Ovary | 1 | 17 | 50 | 16 | 3 | 16 | 24 | 23 | |
| Central nervous system | 13 | 21 | 25 | 16 | 23 | 33 | 30 | 32 | |
| Adrenals | 2 | 35 | 44 | 31 | 19 | 39 | 50 | 62 | |
| Stomach | 3 | 21 | 17 | 14 | 3 | 11 | 8 | 10 | |
| Pancreas | 1 | 6 | 17 | 9 | 10 | 11 | 30 | 18 | |
| Kidney | 2 | 9 | 9 | 7 | 5 | 14 | 19 | 18 | |
| Uterus | 3 | 24 | 9 | 18 | 5 | 17 | 12 | 17 | |
| Thyroid | 2 | 3 | 8 | 16 | 10 | 31 | 22 | 42 | |

(–) Absence of metastases; (+) Presence of metastases.
[a] Number of patients in each of the 8 possible combinations of occurrence at major sites.

TABLE III.—*Sums of Squares Performed on Table II of the Effect of 3 Major Metastatic Sites (Lungs, Liver, Bones) and Related Interactions on the Frequency of Metastases at Minor Metastatic Sites*

| | Minor metastatic sites | | | | | | |
|---|---|---|---|---|---|---|---|
| Effect | Pituitary | CNS | Stomach | Pancreas | Kidney | Uterus | Thyroid |
| Lungs (A) | 0·0141 | 0·0231 | 0·0066 | 0·0162 | 0·0105 | 0·0001 | 0·0722 |
| Liver (B) | 0·0052 | 0·0021 | 0·0015 | 0·0265 | 0·0066 | 0·0006 | 0·0221 |
| Bones (C) | 0·0604 | 0·0015 | 0·0078 | 0·0025 | 0·0021 | 0·0276 | 0·0313 |
| A×B | 0·0001 | 0·0000 | 0·0001 | 0·0008 | 0·0021 | 0·0006 | 0·0002 |
| A×C | 0·0043 | 0·0021 | 0·0003 | 0·0008 | 0·0001 | 0·0021 | 0·0198 |
| B×C | 0·0069 | 0·0078 | 0·0091 | 0·0085 | 0·0045 | 0·0045 | 0·0005 |
| A×B×C | 0·0000 | 0·0010 | 0·0028 | 0·0000 | 0·0000 | 0·0003 | 0·0008 |
| Total | 0·0909 | 0·0376 | 0·0282 | 0·0553 | 0·0259 | 0·0358 | 0·1400 |

TABLE IV.—*Percentages of the Total Variation on the Frequency of Metastases at Minor Metastatic Sites for the Main Effect and Interactions of 3 Major Metastatic Sites (Lungs, Liver, Bones) and Related Interactions**

| | Minor metastatic sites | | | | | | |
|---|---|---|---|---|---|---|---|
| Effect | Pituitary | CNS[b] | Stomach | Pancreas | Kidney | Uterus | Thyroid |
| Lungs (A) | 15 | 61 | 23 | 29 | 41 | 0·3 | 52 |
| Liver (B) | 6 | 6 | 5 | 48 | 25 | 3 | 16 |
| Bones | 66 | 4 | 28 | 5 | 8 | 77 | 22 |
| A × B | 0·1 | 6 | 0·4 | 1 | 8 | 2 | 0·1 |
| B × C | 5 | 21 | 1 | 1 | 0·4 | 6 | 9 |
| A × C | 8 | 3 | 32 | 16 | 17 | 13 | 0·4 |
| A × B × C | 0·0 | 0·0 | 10 | 0·0 | 0·0 | 0·8 | 0·6 |

* This table was obtained from Table III, by dividing each entry by its column total.
[b] Central nervous system.

TABLE V.—*F Values from Analysis of Variance Performed on Table III (Pooled Error with 28 D.F. and 1 D.F. for Each Main Effect)*

| | Minor metastatic sites | | | | | | |
|---|---|---|---|---|---|---|---|
| Effect of major site | Pituitary[a] | CNS[b] | Stomach | Pancreas | Kidney | Uterus | Thyroid |
| Lungs | 5·38* | 8·88† | 2·54 | 6·23† | 4·04 | 0·04 | 27·76† |
| Liver | 2·00 | 0·81 | 0·57 | 10·19† | 2·54 | 0·23 | 8·50† |
| Bones | 23·23† | 0·58 | 3·00 | 0·96 | 0·81 | 10·62† | 12·03† |

* F probability $< 0·05$.
† F. probability $\sim 0·01$.
[a] And/or parathyroid.
[b] Central nervous system.

sites seems to be paramount. Examples are bones for the pituitary (0·19), liver for ovary (0·50), liver and bones for adrenals, liver and lungs for pancreas and bones for uterus.

Table II was analysed as a $2^3$ factorial design whose sums of squares are shown in Table III. The total sum of squares was calculated for each column. Table IV was obtained by dividing each entry in Table III by the column total.

The highest percentage of the total variation for the pituitary gland is due to the bones (0·66) followed by the lungs (0·15). When metastases are present in the central nervous system, 0·61 of the total variation is attributable to the lungs and a (liver × bones) interaction term accounts for 0·21 of the total variation.

The stomach shows a rather erratic pattern; the liver effect explains almost one half (0·45) of the pancreas variation, followed by the lungs effect; the opposite holds true for kidney; the relationship between uterus and bones seems to be paramount (0·77) and the thyroid seems to be more often related to the lungs (0·52), followed by the bones (0·22) and liver (0·16). With the exception of thyroid (Table V), no site has significant F values for all 3 main effects.

The pituitary gland shows 2 significant F values due to the lung and bone " effects ". The central nervous system is related to the lungs; pancreas has a double association, *i.e.* with lungs and liver, the uterus is significantly related to the bony system; F values for the kidney are significant, although the biggest effect is due to the lungs, the second one to the liver and the third one, due to the bones, is negligible. The stomach is quite similar to the kidney, with the only exception of the " liver effect " which appears to be negligible.

### DISCUSSION

Because lungs, liver and bones have a high frequency of metastases, it is not possible to say whether these 3 major metastatic sites are independently seeded by the primary tumour, or whether they influence each other. It seems unlikely, however, that less frequently seeded sites produce metastases in the lungs, liver and bones (Table II).

There are two interpretations of the term " effect ", used in the factorial design: the " single effects " of the factorial design could be interpreted either as a real dissemination of metastases from major sites to minor ones, or as a measure of the strength of the association between 2 organs seeded by the primary tumour. Indeed, autopsy findings do not indicate any time sequence related to the appearance of metastases in different organs. If, for example, lung metastases do not shorten the life span of a patient excessively, the " lung effect " on pancreas would be just a " time effect " long enough so that metastases can reach the pancreas and can therefore proliferate for a long enough period to be detected.

If such an explanation were true, one would expect everywhere in Table V a pattern like that shown by the thyroid, where all the 3 single effects are significant at below 5% probability level. Instead, Table V shows that the pattern of the significant F values is different for different secondary sites. The pattern can be interpreted either in terms of an anatomical proximity, such as liver and pancreas, or in terms of a " blood vessel " metastatic dissemination, such as the lungs and the central nervous system. Either route strongly supports the hypothesis of a multistep dissemination.

It appears (Table V) that the lungs tend to release metastases into the circulatory system (distant metastases), such as the central nervous system, pancreas, pituitary and thyroid. An intra-abdominal, probably lymphatic dissemination of metastases, from the liver to the pancreas, is plausible (multistep lymphatic). The relationship between liver and thyroid is also rather obscure.

The osseous system seems to affect various sites, a few of which are related only to the bones, *i.e.* uterus, and a few others are in common with the other 2 major sites, *i.e.* pituitary and/or parathyroid, and thyroid. This seems to imply, first of all that the osseous system can release metastases into the blood stream which can affect various organs without any filtering effect exerted by the lungs, *i.e.* with no appearance of metastases in the lungs. The osseous system was subdivided into 2 parts—vertebrae (64·9) and others (55·8); therefore, a certain ambiguity might arise in dealing with bones and related peripheral sites because it was not specified which bone was affected by metastases. For kidneys and stomach, none of the F values were significant at the 5% probability level; this simply means that it is not possible to establish any route of dissemination of the tumour to the two aforementioned peripheral organs with these data.

Table IV is in good agreement with the results of Table V. There is a satisfactory correspondence between the significant F values and the highest proportions of the total variation due to single effects (Table IV).

### GENERAL REFERENCES

ADAIR, F. E. & HERRMANN, J. B. (1946) Unusual Metastatic Manifestations of Breast Carcinoma; Metastasis to the Mandible with a Report of Five Cases. *Surg., Gynec. Obstet.*, **83**, 289.

ALDRETE, J. S. & BOHROD, M. G. (1967) Adrenal Metastases in Cancer of the Breast. *Am. Surg.*, **33**, 174.

ASCH, M. J., WIEDEL, P. D. & HABIF, D. V. (1968) Gastrointestinal Metastases from Carcinoma of the Breast. *Archs Surg.*, **96**, 840.

BENELLI, A. (1962) Carcinoma Mammario e Metastasi Uterina. *Minerva ginec.*, **14**, 827.

FITZWILLIAMS, D. C. L. (1925) Carcinoma of the Breast and its Method of Spread: Embolism or Permeation. *Br. J. Surg.*, **12**, 650.

HARTMANN, W. H. & SHERLOCK, P. (1961) Gastroduodenal Metastases from Carcinoma of the Breast. *Cancer, N.Y.*, **14**, 426.

JACOX, R. F. & TRISTAN, T. A. (1960) Carcinoma of the Breast Metastatic to the Bones of the Foot. *Arthritis Rheum.*, **3**, 170.

LENZ, M. & FREID, J. R. (1931) Metastases to the Skeleton, Brain, and Spinal Cord from Cancer of the Breast and the Effect of Radiotherapy. *Ann. Surg.*, **93**, 278.

MCDONALD, J. J., HAAGENSEN, C. D. & STOUT, A. P. (1953) Metastasis from Mammary Carcinoma to the Supraclavicular and Internal Mammary Lymph Nodes. *Surgery, St Louis*, **34**, 521.

SONG, J. (1963) Metastatic Carcinoma of the Uterine Cervix from Primary Breast Cancer. *J. Am. med. Ass.*, **184**, 498.

STALEY, C. J. (1956) Skeletal Metastases in Cancer of the Breast. *Surg., Gynec. Obstet.*, **102**, 683.

STILES, H. J. (1899) On the Dissemination of Cancer of the Breast. *Br. med. J.*, i, 1452.